# Prediction of Oral Intake at Discharge with Early Assessment of Swallowing Function within 24 h after Admission: A Retrospective Cohort Study

**DOI:** 10.1007/s00455-024-10699-x

**Published:** 2024-04-01

**Authors:** Daisuke Matsuura, Yohei Otaka, Saki Asaumi, Tomomi Itano, Tetsushi Chikamoto, Shigeru Yamori, Yusuke Murakami

**Affiliations:** 1 Department of Rehabilitation, Brain Attack Center Ota Memorial Hospital, Hiroshima, Japan; 2 Department of Rehabilitation Medicine I, School of Medicine, Fujita Health University, 1-98 Dengakugakubo, Kutsukake, Toyoake, Aichi 470-1192 Japan; 3 Department of Rehabilitation, Fukuyama rehabilitation hospital, Hiroshima, Japan

**Keywords:** Cerebrovascular disorder, Deglutition disorders, Nutrition, Mann assessment of swallowing ability, Outcome

## Abstract

Dysphagia is one of the most common symptoms of stroke and can lead to other complications such as pneumonia, dehydration, and malnutrition. This retrospective cohort study evaluated the predictive value of a comprehensive swallowing assessment tool, the Mann Assessment of Swallowing Ability (MASA), in the acute phase of stroke for oral intake status at discharge. Among 1,133 consecutive patients with acute stroke, 512 patients whose swallowing function was assessed using the MASA within 24 h of admission were included. Data including demographic information, stroke severity, MASA, Oral Health Assessment Tool, body mass index, and serum albumin level were collected. Predictive factors for oral intake were analyzed using a multiple logistic regression model, and the receiver operating characteristic (ROC) curve analysis was used to determine the cutoff values of the MASA score for determining oral intake at discharge. Oral intake at discharge was established in 69.1% of the cohort (354/512). The multiple logistic regression analysis identified a higher MASA score, younger age, and higher serum albumin level as significant predictors of oral intake at discharge. The cutoff value of the MASA score for oral intake was 136.5 points, with an area under the ROC curve of 0.87. These findings suggest that the MASA is a valid tool for predicting oral intake in patients with dysphagia during the acute phase of stroke.

**Supplementary Information:**

The online version contains supplementary material available at 10.1007/s00455-024-10699-x.

## Introduction

Dysphagia occurs in 30–80% of patients with cerebrovascular injuries [1–3]. Notably, it can disrupt oral intake, leading to complications such as dehydration, malnutrition, and pneumonia [4]. Furthermore, various indicators and factors that may predict the long-term outcomes of oral intake have been investigated and reported, including both direct indicator of swallowing function and other. Direct indicators of swallowing function include dysphagia severity [5], video-fluoroscopy (VF) [6, 7] and video-endoscopy (VE) [8]. Other factors associated with oral intake include age [9], stroke severity [9, 10], consciousness disturbance [11], premorbid independence [10], oral status [12], low body mass index (BMI) [11], and low serum albumin level [12].

Among them, the pathophysiology and severity of dysphagia directly related to the outcome of oral intake, and the benefit of accurate assessment before administration of oral intake has been elucidated [13]. Notably, several dysphagia evaluation methods have been established, including the water swallow test (WST) [14] and VF and VE swallowing examinations [3, 15]. However, assessments using water or food can cause aspiration, and tests requiring certain equipment are not suitable for all patients.

The Mann Assessment of Swallowing Ability (MASA) [16] was developed to comprehensively examine swallowing ability in patients with acute stroke. It includes evaluating cognitive, communicative, respiratory, and motor functions that may influence swallowing. Therefore, it can be scored even in patients with severe dysphagia who are at high risk for the assessment. A previous study used MASA to assess swallowing in relatively few patients with stroke and investigated the association between MASA scores and outcomes for each stroke type [17]; however, to our knowledge, no study is available on the prediction of swallowing outcomes using the MASA in a large number of consecutive patients with acute stroke. Therefore, in this study, we aimed to investigate whether MASA scores during the acute phase of stroke could predict swallowing outcomes in terms of oral intake.

## Methods

### Study Design and Setting

This retrospective cohort study was conducted at Brain Attack Center Ota Memorial Hospital, a stroke center that treats > 1,200 patients with stroke annually. The study protocol was approved by the Medical Research Ethics Review Committee of Ota Memorial Hospital (No. 270) and is reported in accordance with the STROBE guidelines [18]. The requirement for informed consent was waived because this study was retrospective in design and based on the data already in clinical use. Only individuals who did not opt out of the use of their data in our research were included.

### Participants

The participants of this study were consecutive patients with acute cerebral hemorrhage and infarction admitted to the Stroke Care Unit of our hospital between June 2020 and April 2021. All participants underwent magnetic resonance imaging, computed tomography, or both to confirm stroke diagnosis. This study included those suspected of having swallowing problems by the attending physician or physiatrist and whose swallowing function was evaluated using the MASA within 24 h of admission. Among these patients, the following patients were excluded: Those who died before discharge, those admitted to the hospital after > 7 days from the stroke onset, and those aged < 18 years.

### Data Collection

The following clinical data were collected from the patients’ electronic medical records: age, sex, type of stroke, National Institutes of Health Stroke Scale (NIHSS) score on admission as an assessment of stroke severity [19], past history of stroke, and length of hospital stay. Stroke type and NIHSS score were assessed by a board-certified neurologist or neurosurgeon on admission. We also collected data on BMI and serum albumin levels on admission as nutritional indices and the Oral Health Assessment Tool (OHAT), which is reliable and valid for assessing oral health [20]. The MASA [16] was used to assess dysphagia on admission, and the Food Intake Level Scale (FILS) [21] was used to evaluate oral intake status at admission and discharge.

### The MASA

The MASA consists of 24 items related to the swallowing function from the pre-oral to pharyngeal phases. The total score ranges from 38 to 200 points, with higher scores indicating better swallowing function. The total scores classify dysphagia severity into four categories: 187–200, no abnormality; 168–186, mild; 139–167, moderate; and ≤ 138, severe. The reliability and validity of the MASA have already been established [16]. When the attending physician or physiatrist suspects dysphagia in a patient, a speech-language-hearing therapist (ST) promptly evaluates the MASA score within 24 h of admission. Eight STs were responsible for the evaluation during the study period. They received at least 2 months of on-the-job training, and STs with > 5 years of experience cross-checked the data.

### FILS

FILS is a 1–10 scale that measures the degree of daily oral food intake. Levels 1–3 are associated with varying degrees of non-oral feeding. Levels 4–6 are associated with varying degrees of oral food intake and alternative nutrition. Levels 7–9 are associated with varying oral food intake alone, whereas level 10 is associated with normal oral food intake [21]. The scale details are presented in the supplementary table.

### Swallowing Rehabilitation

After admission to the stroke care unit, the indication for swallowing evaluation and rehabilitation for each patient was judged by his/her attending physician or physiatrist. In principle, ST performed the MASA evaluation and rehabilitation in all patients. Based on dysphagia severity or the clinical course, including dietary status, VF or VE indication was decided by the discussion among the attending physician, physiatrist, and ST.

### Analyses

Dysphagia severity on admission was classified into four levels based on the MASA scores, and descriptive statistics were performed based on the FILS score on admission and discharge. Furthermore, the participants were divided into two groups based on their oral intake status at discharge: those who had established oral intake for three meals (FILS ≥ 7) and those who had not (FILS ≤ 6). We compared the clinical factors between these groups using Pearson’s chi-square and Mann–Whitney U tests, depending on the variable type. A multiple logistic regression analysis with the MASA score was performed to achieve oral intake at discharge (FILS ≥ 7), adjusted for age and those variables that were significantly different in between-group comparisons. Finally, receiver operating characteristic (ROC) analysis of the MASA score for oral intake at discharge was performed. The cutoff value was determined by the largest Youden index (sensitivity + specificity − 1) [22]. The sensitivity, specificity, and area under the ROC curve (AUC) were also determined. In addition, we conducted the same analyses as a sub-analysis on the group of patients who were not independent in oral intake on admission. All statistical analyses were performed using the IBM SPSS Statistics Ver. 24.0 software (IBM Corporation, Armonk, NY, USA). Statistical significance was set at P values < 0.05.

## Results

**Fig. 1:**
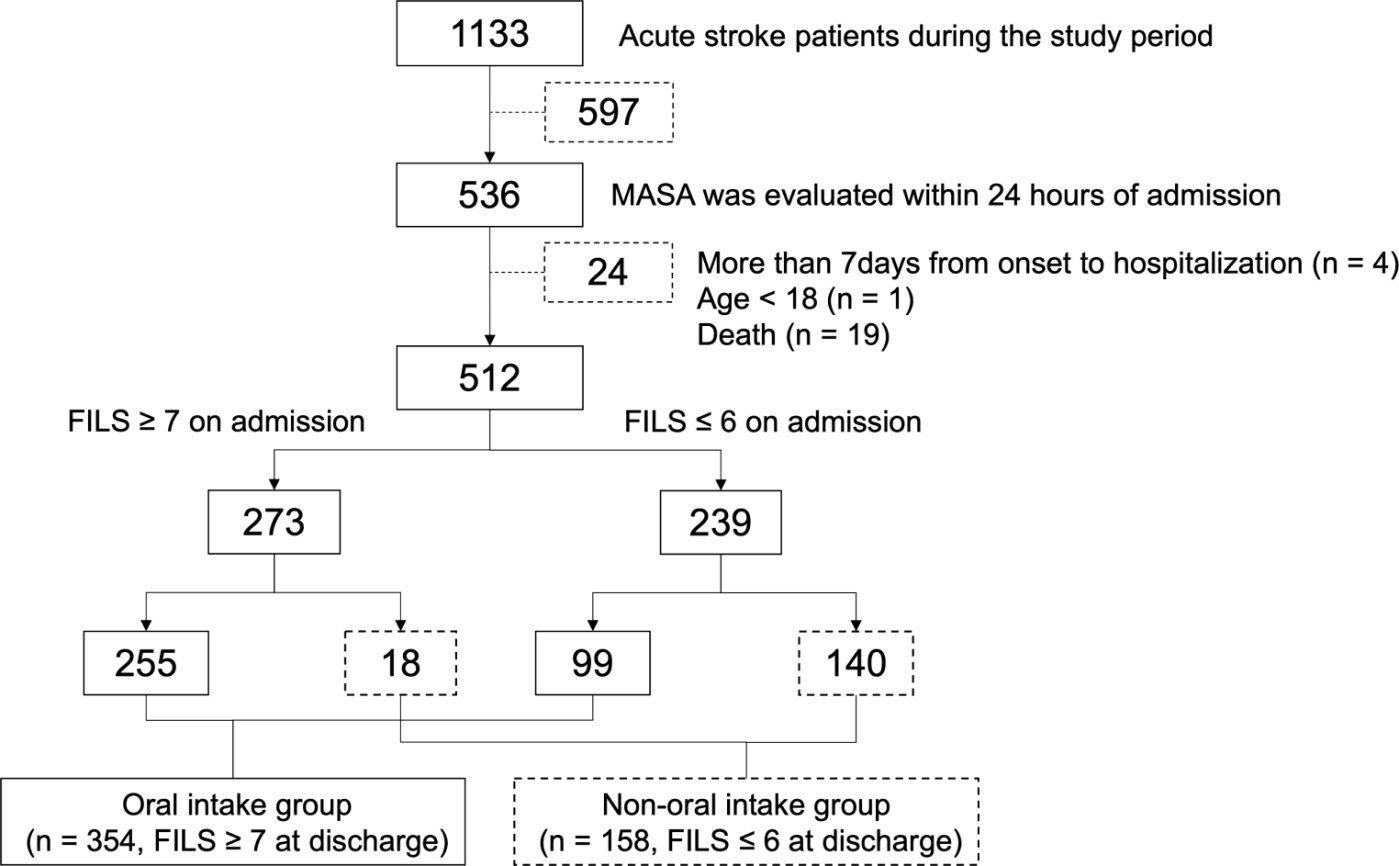
Flow chart to show cohort inclusion in the study. FILS, Food Intake LEVEL Scale

**Fig. 2:**
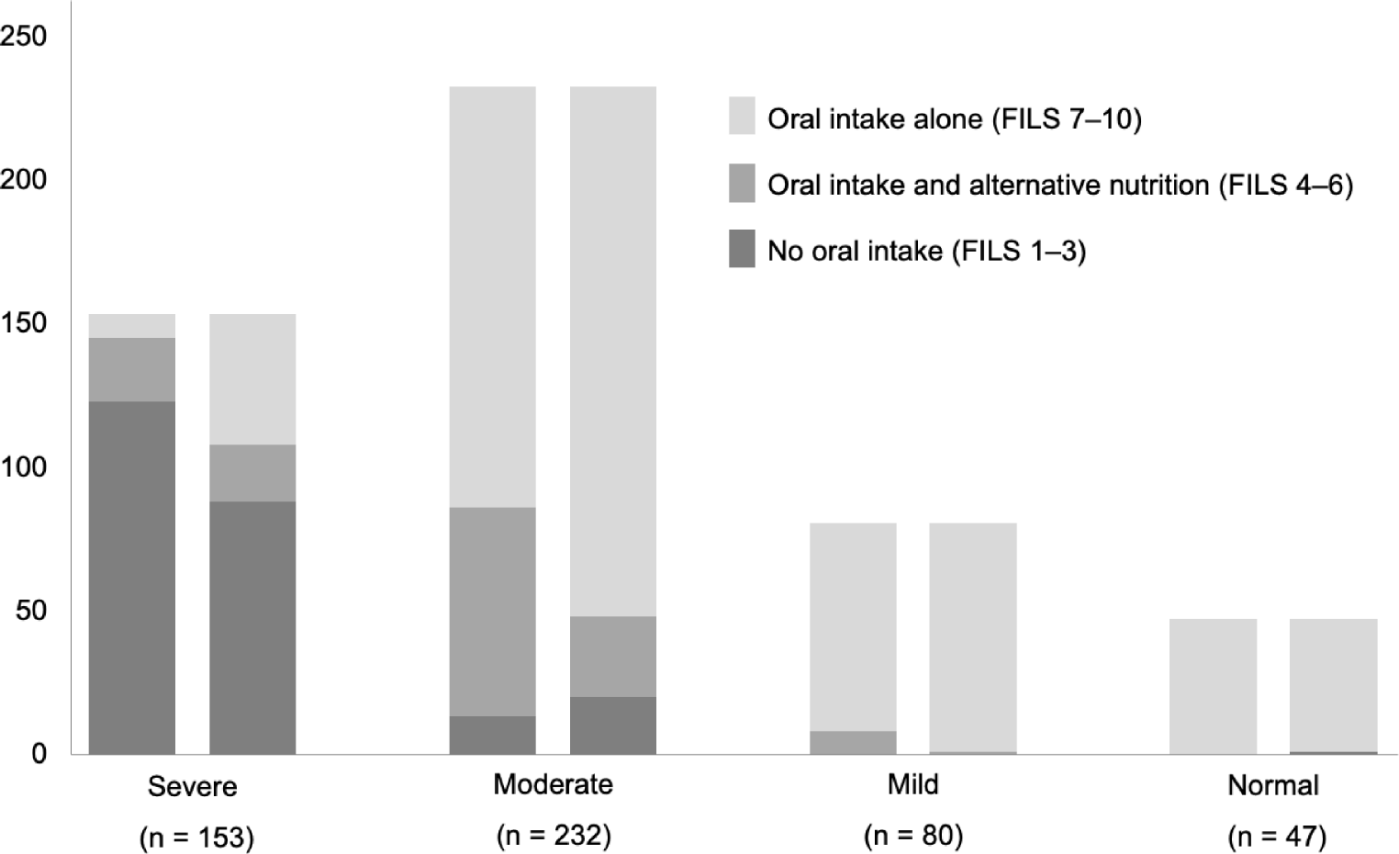
Distribution of the Food Intake Level Scale (FILS) scores, based on dysphagia severity, evaluated using the Mann Assessment of Swallowing Ability (MASA) on admission and discharge Left and right bars in each severity indicate data on admission and discharge, respectively

Among the 1,133 patients with acute stroke admitted during the study period, 512 who fulfilled the criteria were enrolled in the study (Fig. 1). The mean (standard deviation) age of the patients was 78.0 (11.2) years; 386 patients had cerebral infarction, and 126 had a cerebral hemorrhage, with a median (interquartile range) NIHSS score of 9 (4–20) on admission (Table 1). The percentage of patients who were independent in oral intake (FILS ≥ 7) on admission was 53.3% (*n* = 273), whereas that of those at discharge was 69.1% (*n* = 354). Among 239 patients who were not independent in oral intake (FILS ≤ 6) on admission, 99 (41.8%) achieved independent oral intake at discharge (Fig. 1). Dysphagia severity on admission, based on MASA score, was severe in 153 patients, moderate in 232 patients, mild in 80 patients, and normal in 47 patients. Figure 2 shows the oral intake status of each MASA severity group on admission and discharge. The MASA severity was inversely correlated with the percentage of patients with oral intake independence (FILS ≥ 7); 94.8% of the patients categorized as having severe dysphagia were not independent in oral intake on admission, and 70.6% still had not become independent at discharge.

**Table 1:** Characteristics of all the patients and comparisons between oral intake and non-oral intake groups

	All patients *n* = 512	Oral intake group (FILS ≥ 7 at discharge) *n* = 354	Non-oral intake group (FILS ≤ 6 at discharge) *n* = 158	P value
Age, years, mean (SD)	79.1 (11.1)	78.0 (11.5)	81.7 (9.6)	< 0.001
Sex, male/female, n (%)	266/256 (52.0/48.0)	195/159 (55.1/44.9)	71/87 (44.9/55.1)	0.034
BMI, kg/m^2^, mean (SD)	22.4 (4.2)	22.6 (3.9)	21.7 (4.6)	0.002
BMI, classification, n (%) Underweight (BMI < 18.5) Normal weight (18.5 ≤ BMI < 25) Overweight (25 ≤ BMI < 30) Obesity (30 ≤ BMI)	81 (15.8) 313 (61.1) 95 (18.6) 23 (4.5)	50 (14.1) 209 (59.0) 77 (21.8) 18 (5.1)	31 (19.6) 104 (65.8) 18 (11.4) 5 (3.2)	0.018
Albumin, g/dl, mean (SD)	3.6 (0.6)	3.8 (0.5)	3.4 (0.7)	< 0.001
Type of stroke, infarction/hemorrhage, n (%)	386/126 (75.4/24.6)	283/71 (79.9/20.1)	103/55 (65.2/34.5)	< 0.001
History of stroke, n (%)	169 (33.0)	119 (33.6)	50 (31.6)	0.661
MASA score on admission, median (IQR)	155 (130–167)	162 (151–172)	119 (91–145)	< 0.001
NIHSS score on admission, median (IQR)	9 (4–20)	6 (3–12)	20 (12–28)	< 0.001
OHAT score on admission, median (IQR)	0 (0–2)	0 (0–2)	1 (0–1)	0.003
Length of hospitalization, days, mean (SD)	18.6 (9.7)	17.1 (8.3)	21.8 (11.7)	< 0.001

*FILS* Food Intake LEVEL Scale, *SD* standard deviation, *BMI* body mass index, *MASA* Mann Assessment of Swallowing Ability, *NIHSS* National Institutes of Health Stroke Scale, *OHAT* Oral Health Assessment Tool, *IQR* interquartile range

Patients who were receiving oral intake at discharge (oral intake group, FILS ≥ 7) were compared to those who were not receiving oral intake at discharge (non-oral intake group, FILS ≤ 6). The oral intake group was significantly younger and had a lower NIHSS score and a higher MASA score than the non-oral intake group. The non-oral intake group was also significantly more likely to be underweight based on the BMI classification, have lower albumin levels, and have poorer oral hygiene (higher OHAT score) (Table 1). The patients who were not independent in oral intake (FILS ≤ 6) on admission but were in the oral intake group at discharge had a significantly lower NIHSS score and a higher MASA score than those in the non-oral intake group at discharge. In addition, the non-oral intake group at discharge was significantly more likely to be underweight and had lower albumin levels and poorer oral hygiene (higher OHAT score) (Table 2).

**Table 2:** Characteristics of patients who were not independent of oral intake (FILS ≤ 6) on admission and comparison between oral intake and non-oral intake groups

	All patients with FILS ≤ 6 on admission *n* = 239	Oral intake group (FILS ≥ 7 at discharge) *n* = 99	Non-oral intake group (FILS ≤ 6 at discharge) *n* = 140	P value
Age, years, mean (SD)	80.4 (11.4)	78.5 (13.2)	81.9 (9.7)	0.111
Sex, male/female, n (%)	107/132 (44.8/55.2)	48/51 (48.5/51.5)	59/81 (42.1/57.9)	0.331
BMI, kg/m^2^, mean (SD)	21.9 (3.9)	22.5 (4.3)	21.5 (3.6)	0.025
BMI, classification, n (%) Underweight (BMI < 18.5) Normal weight (18.5 ≤ BMI < 25) Overweight (25 ≤ BMI < 30) Obesity (30 ≤ BMI)	47 (19.7) 141(59.0) 43 (18.0) 8 (3.3)	17(17.2) 52 (52.5) 26 (26.3) 4 (4.0)	30 (21.4) 89 (63.6) 17 (12.1) 4 (2.9)	0.038
Albumin, g/dl, mean (SD)	3.4 (0.7)	3.6 (0.6)	3.3 (0.7)	0.010
Type of stroke, infarction/hemorrhage, n (%)	159/80	69/30 (69.7/30.3)	90/50 (64.3/35.7)	0.383
History of stroke, n (%)	80 (33.8)	36 (36.4)	44 (31.4)	0.426
MASA score on admission, median (IQR)	128 (98–147)	143 (128–158)	111 (85–136)	< 0.001
NIHSS score on admission, median (IQR)	18 (8–26)	12.0 (5.0–20.0)	21.5 (12.3–28.0)	< 0.001
OHAT score on admission, median (IQR)	1 (0–2)	0 (0–2)	1 (0–3)	0.018
Length of hospitalization, days, mean (SD)	21.4 (12.1)	20.1 (12.1)	22.0 (12.2)	0.018

*FILS* Food Intake LEVEL Scale, *SD* standard deviation, *BMI* body mass index, *MASA* Mann Assessment of Swallowing Ability, *NIHSS* National Institutes of Health Stroke Scale, *OHAT* Oral Health Assessment Tool, *IQR* interquartile ratio

**Fig. 3:**
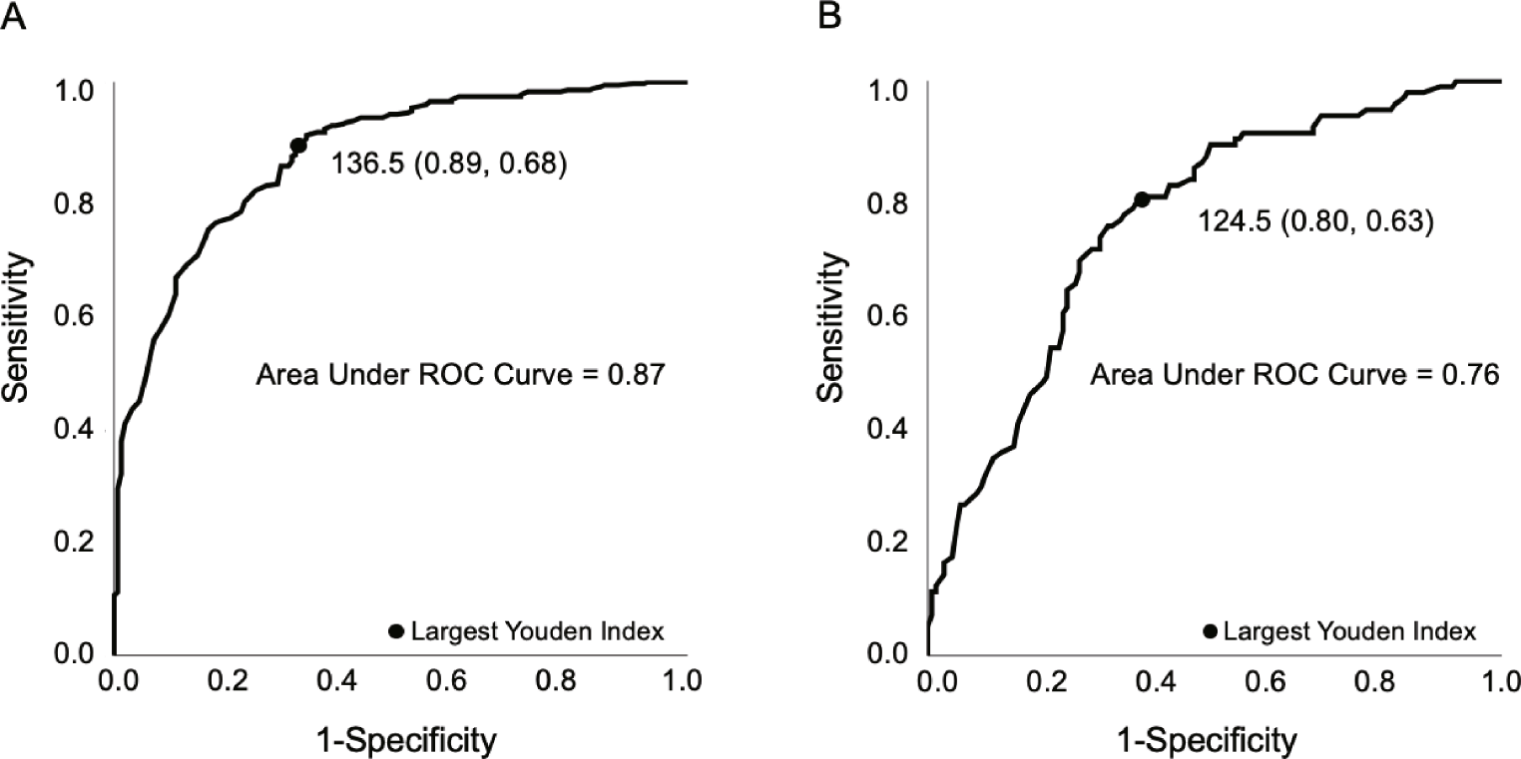
Receiver operating characteristic (ROC) curves of the Mann Assessment of Swallowing Ability (MASA) score for oral intake independence. (**A**) All patients (*n* = 512) and (**B**) patients who were not independent (FILS ≤ 6) on admission (*n* = 239). Independence in oral intake was defined as FILS ≥ 7. FILS, Food Intake Level Scale

We performed a multiple logistic regression analysis adjusted for age and variables associated with oral intake at discharge in the univariate analyses. The analysis revealed that the MASA score was significantly associated with oral intake status at discharge and that age and serum albumin level on admission were also significantly associated (Table 3). In the ROC curve, the AUC was 0.87 (Fig. 3a). The cutoff value was 136.5, and sensitivity and specificity were 0.89 and 0.68, respectively.

**Table 3:** Multiple logistic analysis of all the patients for prediction of oral intake at discharge

	Odds ratio	95% Confidence interval	P value
Age, years	-0.04	0.93–0.99	0.006
Sex, male	-0.22	0.47–1.36	0.411
MASA score on admission	1.05	1.03–1.06	< 0.001
NIHSS score on admission	-0.03	0.94–1.01	0.105
OHAT score	-0.06	0.81–1.08	0.380
Body mass index, kg/m^2^	0.01	0.94–1.08	0.815
Albumin, g/dl	0.55	1.13–2.68	0.012
Cerebral infarction	0.30	0.74–2.58	0.304

*MASA* Mann Assessment of Swallowing Ability, *NIHSS* National Institutes of Health Stroke Scale, *OHAT* Oral Health Assessment Tool

Similarly, in the multiple regression analysis restricted to patients who were not independent in oral intake (FILS ≤ 6) on admission, the MASA score and age on admission were significantly associated with oral intake status at discharge (Table 4). In the ROC curve, the AUC was 0.76 (Fig. 3b). The cutoff value was 124.5, and sensitivity and specificity were 0.80 and 0.63, respectively.

**Table 4:** Multiple logistic analysis of the patients who were not independent (FILS ≤ 6) on admission for prediction of oral intake at discharge

	Odds ratio	95% Confidence interval	P value
Age, years	-0.04	0.94–1.00	0.021
MASA score on admission	0.03	1.02–1.05	< 0.001
NIHSS score on admission	-0.01	0.95–1.03	0.546
OHAT score	-0.14	0.73–1.02	0.091
Body mass index, kg/m^2^	0.01	0.93–1.09	0.901
Albumin, g/dl	0.33	0.87–2.24	0.165

*MASA* Mann Assessment of Swallowing Ability, *NIHSS* National Institutes of Health Stroke Scale, *OHAT* Oral Health Assessment Tool

Supplementary table. Food Intake LEVEL Scale (FILS)

## Discussion

This study aimed to identify the prognostic factors for oral intake in acute stroke. The multiple logistic regression analysis identified higher MASA scores, younger age, and higher serum albumin levels as significant predictors of oral intake at discharge. This study used the MASA to assess swallowing function in patients with acute stroke early after admission. The results suggest that the MASA score within 24 h of admission in the acute phase of stroke is a powerful predictor of oral intake outcome, even after adjusting for other potentially related factors such as age, stroke severity, and nutritional status. The present study showed for the first time that the MASA can be used as a prognostic tool as well as a comprehensive assessment tool for swallowing function in patients with acute stroke.

Previous studies have investigated the association between oral intake and various types of initial swallowing assessments, including bedside swallowing tests [23, 24] and particular dysphagia indices [23] in patients with acute stroke. WST, a simple bedside assessment of swallowing, was not a significant factor for oral intake, although it has been validated as a predictor of aspiration pneumonia [23, 24]. Furthermore, it has a low specificity for detecting aspiration [24], and silent aspiration may be missed [14]. Regarding the study using index for dysphagia, Broadley et al. reported that a standardized assessment using the Parramatta Hospitals Assessment of Dysphagia score within 72 h of admission was significantly associated with prolonged dysphagia [23]. The study was conducted in a between-group comparison and did not provide cutoff values for predicting oral intake. It is difficult to directly compare the present study with these studies; however, MASA was an independent predictor of prognosis, and a clear cutoff value was demonstrated in the present study.

Swallowing examinations using instruments have been reported to have high predictive accuracy. Ickenstein et al. reported that in patients with acute stroke, signs of aspiration confirmed by clinical swallowing examination within 24 h and the penetration–aspiration scale (PAS) ≥ 5 by VE within 72 h had a high predictive value for failure to resume oral intake at 90 days (odds ratio 11.8, 95% confidence interval 0.036–0.096). Han et al. reported that a 14-item quantitative scale of VF findings (Video-fluoroscopic dysphagia scale: VDS) was useful as a predictor of long-term prognosis after stroke (cutoff value of 47, sensitivity was 0.91, and specificity was 0.92) [6]. Therefore, it may be necessary to consider the effective use of instrumental assessment in combination with the MASA to achieve a high predictive accuracy consistent with these results.

In addition to swallow-specific assessments, the relationship between overall stroke severity and swallowing outcome has also been investigated. The NIHSS, widely used in the acute phase to assess stroke severity, has also been discussed with regards to dysphagia prognosis. Galovic et al. reported a prognostic model including these variables: age, NIHSS on admission, lesion location, initial aspiration risk, and initial impairment of oral intake, which showed discrimination (C statistic) of 0.77 (95% CI, 0.67–0.87; *P* < 0.001) in predicting oral intake recovery at day 30 [9]. However, Nakajima et al. [10] and Lee et al. [5] reported that the initial NIHSS score was not significantly associated with long-term swallowing function recovery. Similarly, multiple logistic regression analysis in the present study identified the initial NIHSS as a non-significant variable for oral intake. It can be assumed that the MASA, a more swallow-specific assessment, is superior to the NIHSS as an initial assessment for predicting oral intake.

Serum albumin level on admission and age were also significantly associated with oral intake at discharge in the multiple logistic regression analysis. A low albumin level on admission may reflect low nutritional status before stroke onset, and it has also been observed as a relevant factor in predicting the resumption of oral intake in patients with subacute stroke [25]. Regarding age, it is associated with all fundamental human functions, and many studies have reported it as a factor associated with oral intake [26, 27]. Clinicians should consider that, in addition to dysphagia severity, low nutritional status, and age can affect the clinical outcomes of acute stroke dysphagia.

The study’s clinical implication is presenting the MASA score as a possible reference for oral intake. This study identified a MASA score of 136.5 within 24 h of admission as the cutoff value that predicts oral intake at discharge. This value was close to the borderline between severe (≤ 138) and moderate (139–167) MASA dysphagia classifications [16], suggesting it is a vital point in determining oral intake in the acute phase of stroke. If the MASA score is below this point, more careful planning with detailed assessments such as VF is required when aiming for oral intake by discharge; however, if it is above this point, it may provide a basis for planning oral intake resumption. Furthermore, in patients requiring tube feeding after stroke onset, a MASA score of ≥ 124.5 would increase the likelihood of resumption of oral intake. Of course, oral intake is still possible even if the score is below the cutoff, but the MASA score can potentially help the long-term outlook on whether dysphagia will persist.

The present study has some limitations. First, as this was a single-center, retrospective cohort study, the generalizability of our findings to other institutions should be considered cautiously. Second, this study only included the patients who underwent swallowing assessment within 24 h by ST following the physician’s request. Therefore, it is possible that milder or more severe cases were excluded and may not reflect trends in the overall stroke population. In addition, the MASA is sequential from the pre-oral to the pharyngeal phase, so each item reflects a different pathological condition. Further studies are necessary to examine the MASA items in more detail to investigate the appropriate approach and accurate prediction of oral intake for each dysphagia pathology.

In conclusion, this study revealed that the MASA score was significantly associated with the establishment of oral intake at discharge in acute care settings, and thus, the MASA may be a useful assessment tool for dysphagia in acute stroke rehabilitation.

## Electronic supplementary material

Below is the link to the electronic supplementary material.

Supplementary Material 1

## Data Availability

The datasets generated during and/or analysed during the current study are available from the corresponding author on reasonable request.
