# Supplementary material for: Prediction of Oral Intake at Discharge with Early Assessment of Swallowing Function within 24 h after Admission: A Retrospective Cohort Study
Source: Dysphagia. 2024 Apr 1;39(6):1163–70. doi: 10.1007/s00455-024-10699-x (PMC11607090; doi:10.1007/s00455-024-10699-x)
Supplement: Supplementary file 1 — Supplementary Material 1 [file 455_2024_10699_MOESM1_ESM.docx]

Supplementary table. Food Intake LEVEL Scale (FILS)

| No oral intake | |
| --- | --- |
| Level 1  Level 2  Level 3 | No swallowing training is performed except for oral care.  Swallowing training not using food is performed.  Swallowing training using a small quantity of food is performed. |
| Oral intake and alternative nutrition | |
| Level 4  Level 5  Level 6 | Easy-to-swallow food less than the quantity of a meal (enjoyment level) is ingested orally.  Easy-to-swallow food is orally ingested in one to two meals, but alternative nutrition is also given.  The patient is supported primarily by ingestion of easy-to-swallow food in three meals, but alternative nutrition is used as a complement. |
| Oral intake alone | |
| Level 7  Level 8  Level 9  Level 10 | Easy-to-swallow food is orally ingested in three meals. No alternative nutrition is given.  The patient eats three meals by excluding food that is particularly difficult to swallow.  There is no dietary restriction, and the patient ingests three meals orally, but medical considerations are given.  There is no dietary restriction, and the patient ingests three meals orally (normal). |
